# Increased Expression of Micro-RNA-23a Mediates Chemoresistance to Cytarabine in Acute Myeloid Leukemia

**DOI:** 10.3390/cancers12020496

**Published:** 2020-02-20

**Authors:** Stefan Hatzl, Bianca Perfler, Sonja Wurm, Barbara Uhl, Franz Quehenberger, Susanne Ebner, Jakob Troppmair, Andreas Reinisch, Albert Wölfler, Heinz Sill, Armin Zebisch

**Affiliations:** 1 Division of Hematology, Medical University of Graz, Auenbruggerplatz 38, 8036 Graz, Austria; stefan.hatzl@medunigraz.at (S.H.); bianca.perfler@medunigraz.at (B.P.); wurm.sonja1@gmail.com (S.W.); barbara.uhl@medunigraz.at (B.U.); a.reinisch@medunigraz.at (A.R.); albert.woelfler@medunigraz.at (A.W.); heinz.sill@medunigraz.at (H.S.); 2 Institute for Medical Informatics, Statistics and Documentation, Medical University of Graz, 8036 Graz, Austria; franz.quehenberger@medunigraz.at; 3 Daniel Swarovski Research Laboratory, Department of Visceral, Transplant and Thoracic Surgery, Medical University of Innsbruck, 6020 Innsbruck, Austria; susanne.ebner@i-med.ac.at (S.E.); jakob.troppmair@i-med.ac.at (J.T.); 4 Otto-Loewi-Research Center for Vascular Biology, Immunology and Inflammation, Division of Pharmacology, Medical University of Graz, Universitätsplatz 4, 8010 Graz, Austria

**Keywords:** micro-RNA-23a, acute myeloid leukemia, therapeutic resistance, cytarabine

## Abstract

Resistance to chemotherapy is one of the primary obstacles in acute myeloid leukemia (AML) therapy. Micro-RNA-23a (miR-23a) is frequently deregulated in AML and has been linked to chemoresistance in solid cancers. We, therefore, studied its role in chemoresistance to cytarabine (AraC), which forms the backbone of all cytostatic AML treatments. Initially, we assessed AraC sensitivity in three AML cell lines following miR-23a overexpression/knockdown using MTT-cell viability and soft-agar colony-formation assays. Overexpression of miR-23a decreased the sensitivity to AraC, whereas its knockdown had the opposite effect. Analysis of clinical data revealed that high miR-23a expression correlated with relapsed/refractory (R/R) AML disease stages, the leukemic stem cell compartment, as well as with inferior overall survival (OS) and event-free survival (EFS) in AraC-treated patients. Mechanistically, we demonstrate that miR-23a targets and downregulates topoisomerase-2-beta (*TOP2B*), and that *TOP2B* knockdown mediates AraC chemoresistance as well. Likewise, low *TOP2B* expression also correlated with R/R-AML disease stages and inferior EFS/OS. In conclusion, we show that increased expression of miR-23a mediates chemoresistance to AraC in AML and that it correlates with an inferior outcome in AraC-treated AML patients. We further demonstrate that miR-23a causes the downregulation of *TOP2B*, which is likely to mediate its effects on AraC sensitivity.

## 1. Introduction

Acute myeloid leukemia (AML) is an aggressive hematopoietic malignancy and the most common form of acute leukemias in adults [1]. The treatment of AML in younger and/or medically fit patients is based on so-called high dose chemotherapy regimens and hematopoietic stem cell transplantation (HSCT). In more detail, patients are initially treated with one or two chemotherapeutic induction courses, consisting of continuous infusion cytarabine (AraC) over seven days, which is usually combined with three days of an anthracycline, in most cases daunorubicin (7 + 3 scheme). In case a complete remission (CR) is achieved, HSCT and/or intermediate- to high-dose AraC are administered as a consolidation phase [2,3]. The 7 + 3 induction regimen results in CR rates ranging from 60 to 85% in patients younger than 60 years and from 40 to 60% in patients older than 60 years [1,4]. Despite these promising CR rates, a significant subset of patients presents with a primary chemorefractory disease or develops a chemoresistant relapse after CR has been achieved [1,4,5,6,7]. Chemoresistance has been extensively studied in the last years and seems to be multifaceted. It is influenced by a variety of factors, including the tumor burden, growth kinetics, molecular heterogeneity, physical barriers, the immune system, the tumor microenvironment, the persistence of “undruggable” molecular drivers, as well as the consequences of applying therapeutic pressures [8,9,10,11]. In AML, chemoresistance arises quite frequently from an intrinsically chemoresistant leukemic stem cell (LSC) pool, which has the potential to survive chemotherapy, even if the bulk AML is cleared [12,13,14,15,16]. Altogether, chemoresistance is one of the primary reasons for the still dismal survival rates in AML, which range between 35–40% in younger patients, and between 5–15% in patients older than 60 years [1]. Therefore, overcoming chemoresistance is one of the primary targets of AML research with a tremendous potential to increase survival if overcome.

Micro-RNAs (miRs) are short 19–24 nucleotides long RNA fragments. Although not translated into proteins, they play a seminal role in a variety of cellular functions, primarily via regulation of intracellular gene expression levels [17,18,19]. This is orchestrated by inhibition of transcription and translation on the one hand, and by epigenetic modification and destabilization of protein-coding genes, on the other hand [17]. Aberrant miR expression profiles can be detected in the majority of human malignancies, and their functional relevance for the pathogenesis of these diseases is continuously being unraveled [18,20,21,22,23]. miR-23a is a member of the miR-23a/27a/24-2 cluster. It functions as a pivotal regulator of many basic cellular functions, including proliferation, differentiation, and apoptosis [24,25]. Increased expression of miR-23a can be observed in AML, and its functional relevance for myeloid leukemogenesis could be shown by other groups and us recently [26,27,28]. Recent data pinpoint the role of miR-23a in the development of chemoresistance, as increased miR-23a expression decreased the sensitivity to cisplatin and 5-Fluorouracil in a series of solid tumors [29,30,31,32].

In this study, we aimed to delineate whether increased expression of miR-23a in AML affects the chemosensitivity of leukemic cells to AraC, which forms the backbone of AML therapy. We demonstrate that increased expression of miR-23a mediates AraC resistance and that it correlates with an inferior outcome in AraC-treated AML patients. We also demonstrate, that miR-23a is highly expressed at the stage of the primary chemoresistant disease and/or chemoresistant relapse and that it is linked to the intrinsically resistant LSC pool. Mechanistically, we show that DNA topoisomerase 2-beta (*TOP2B*) is a direct target of miR-23a and that *TOP2B* downregulation is likely to mediate the effects of miR-23a on AraC resistance.

## 2. Results

### 2.1. miR-23a Mediates Resistance to AraC

**Figure 1:**
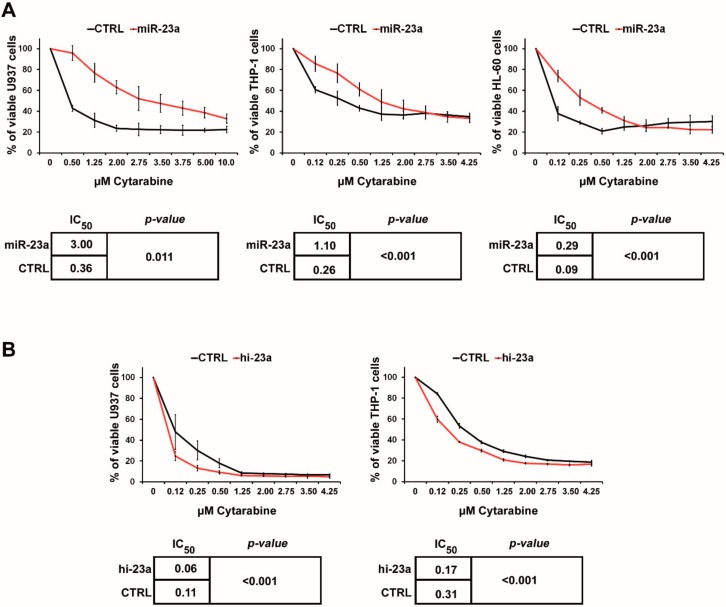
Sensitivity to cytarabine after miR-23a modulation in AML cell lines. (**A**) MTT cytotoxicity assays in AML cell lines after incubation with cytarabine. miR-23a denotes transfection/transduction with a miR-23a overexpression construct; CTRL denotes transfection/transduction with an empty control vector. (**B**) Experiments were repeated in AML cell lines with a knockdown of miR-23a, as achieved by the transfection of miR-23a hairpin inhibitors (hi-23a). Experiments were repeated at least three times. The curves depict the mean ± SD. Statistical significance between IC_50_ values was calculated using Student’s *t*-test.

**Figure 2:**
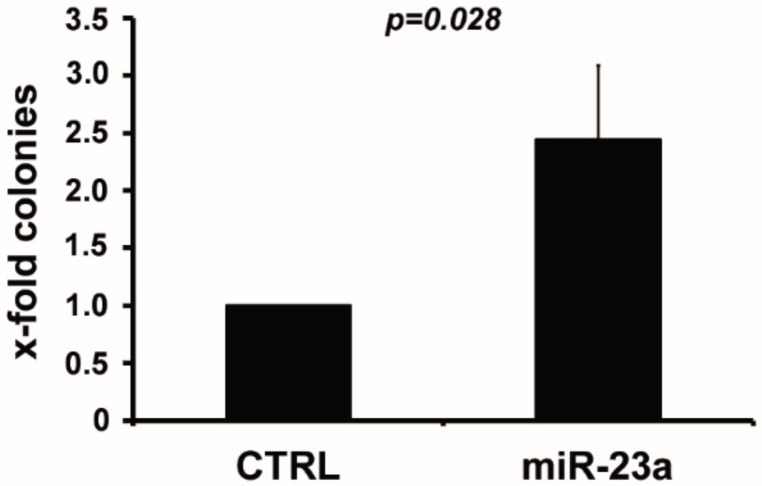
Clonogenic assays after miR-23a overexpression and cytarabine-treatment. For soft agar colony formation assays, U937 cells carrying a miR-23a overexpression construct (miR-23a) and empty vector control (CTRL), respectively, were incubated with 5 µM cytarabine for 2 h. Subsequently, 7500 cells were seeded in 4 mL soft agar and colonies were counted after 8–10 days. The graphs summarize the results of at least three independent experiments. Data are expressed as mean ± SD, and the *p*-value has been calculated using Student’s *t*-test.

We aimed to delineate whether miR-23a affects the sensitivity to AraC, which forms the backbone of cytotoxic AML therapy, and which is not only used during the 7 + 3 induction regimen but also for consolidation in patients who achieved a CR [1,4]. For this purpose, we overexpressed miR-23a in U937 and THP-1 (stable overexpression), as well as in HL-60 (transient overexpression). Subsequently, these cells were incubated with increasing amounts of AraC, which were similar to those encountered in the plasma of AraC-treated AML patients [33]. AraC sensitivity was then assessed in MTT assays. Interestingly, overexpression of miR-23a significantly reduced the sensitivity to AraC in all cell lines tested (Figure 1A). These results could be confirmed by knockdown of miR-23a with hairpin inhibitors. In this case, the sensitivity to AraC was increased in the conditions where miR-23a was knocked down (Figure 1B). Of note, the efficacy of daunorubicin, the most commonly used anthracycline within the 7 + 3 regimen, was not altered in the leukemic cell lines with stable overexpression of miR-23a (Supplementary Figure S1). We then aimed to confirm these data in colony formation assays in semi-solid media supplemented with AraC. These assays provide an essential addition, as they also assess the effects of AraC incubation over a more extended period, an aspect not sufficiently displayed in the short term MTT assays. As only U937 cells demonstrated a sufficient focus forming ability in these assays, we focused on these cells in these experiments. In agreement with the data presented above, miR-23a overexpression caused a significantly increased formation of colonies when compared to the empty vector transduced control cells (Figure 2). Taken together, these data indicate that increased expression of miR-23a mediates resistance to AraC in AML cells. Of note, despite the use of several expression constructs, we were not able to perform a stable knockdown of miR-23a in any of the cell lines studied (data not shown), which prevented the analysis miR-23a downregulation in the long-term colony formation assays.

### 2.2. Increased Expression of miR-23a Correlates with Relapsed/Refractory AML, with the Leukemic Stem Cell Pool and with Shorter Survival in AraC-Treated Patients

**Figure 3:**
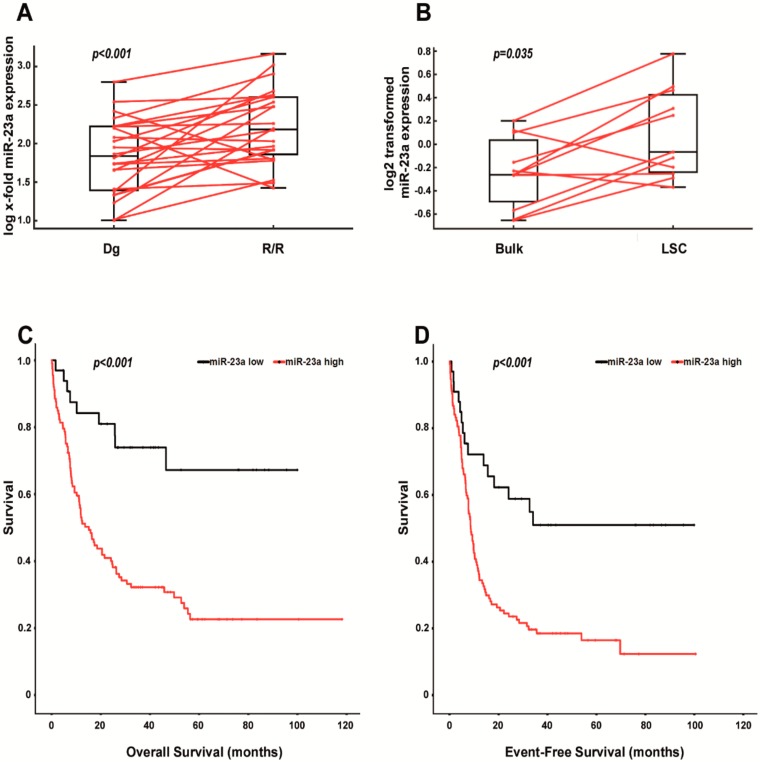
Expression of miR-23a in primary AML patient specimens. (**A**) Box plots displaying miR-23a expression levels in 24 paired AML patient specimens collected at the stage of diagnosis (Dg) and relapsed/refractory disease (R/R). miR-23a expression levels were analyzed by qPCR and are displayed as the log-transformed x-fold expression of the calibrator (NB4 cells). The *p*-value has been calculated using the Wilcoxon signed-rank test. (**B**) Box plots displaying miR-23a expression levels in eleven AML bulk specimens (Bulk) and the corresponding leukemic stem cell (LSC) compartment. Expression data were downloaded from the Gene Expression Omnibus (http://www.ncbi.nlm.nih.gov/geo; accession number GSE55916; Ref [13]) and re-analyzed for the expression of miR-23a. The *p*-value has been calculated using the Wilcoxon signed-rank test. The *y*-axis displays normalized and log2-transformed miR-microarray expression data, as outlined in more detail previously [13]. (**C**,**D**) Overall survival (**C**) and event-free survival (**D**) in AML patients treated with AraC-containing high-dose regimens (n = 146) according to the miR-23a expression status. Data were downloaded from the TCGA-AML dataset [35], and re-analyzed for the expression of miR-23a. Statistical significance was calculated by Cox regression analysis.

In the next step, we aimed to delineate the clinical relevance of these findings. Of note, the chemorefractory disease often develops from an LSC pool [12,13,14,15,16,34]. Even if initial chemotherapy cleared the bulk AML, a small fraction of LSCs might survive this therapy and give rise to a chemoresistant relapse. In this situation, the relapse or primary chemoresistant situation (R/R stage) molecularly resembles the chemorefractory LSC pool. Therefore, we initially analyzed miR-23a expression levels in 24 primary AML patient specimens. All these patients were uniformly treated with 7 + 3 induction and were consolidated with either AraC or HSCT (Supplementary Table S1). Most importantly, however, the material was always obtained at both diagnosis and the R/R stage. Indeed, miR-23a expression was significantly increased at the R/R stage (Figure 3A), which suggests that cells with high miR-23a expression levels represent a chemorefractory subfraction of AML. To delineate, if high miR-23a expression levels truly correlate with the LSC compartment, we re-analyzed a previously published miRNA array data set from Lechman ER et al. [13] via the Gene Expression Omnibus (Series GSE55916). In this study, the authors performed CD34/CD38 sorting from a series of AML patient specimens and tested the leukemic engraftment potential of each subset in NOD/SCID/gamma-null (NSG) mice. Engrafting, leukemia-initiating subsets were considered to contain LSCs. Subsequently, they performed miRNA arrays in each population and calculated an LSC miRNA expression score. In the present study, we focused on specimens, where expression data from both, engrafting CD34/CD38 subsets and corresponding AML bulk material was available (*n* = 11). In agreement with the clinical data presented above, miR-23a expression was significantly increased in populations containing leukemia engrafting LSCs, when compared to the corresponding AML bulk material (Figure 3B).

Finally, we aimed to validate the role of miR-23a in chemoresistance in an independent cohort of AML patients, and therefore, analyzed miR-23a expression levels within the TCGA-AML database, where data about the treatment regimen, as well as about EFS and OS are provided [35]. By focusing on patients treated with AraC-containing high-dose regimens only (*n* = 146), we observed that high miR-23a expression levels correlated statistically significant with shorter EFS and OS within this cohort (Figure 3C; for clinical characteristics of patients see Supplementary Table S2 and Supplementary Figure S2). We then tried to corroborate these results in a multivariate model and, therefore, focused on OS, which is generally viewed as the most stringent parameter in the analysis of biomarkers with a potential predictive/prognostic value. By including the established AML risk factors age at diagnosis, WBC and cytogenetics, we could validate an independent predictive role of miR-23a in AraC-treated patients (Table 1).

### 2.3. TOP2B Is Regulated by miR-23a and Affects the Sensitivity to AraC as Well

**Figure 4:**
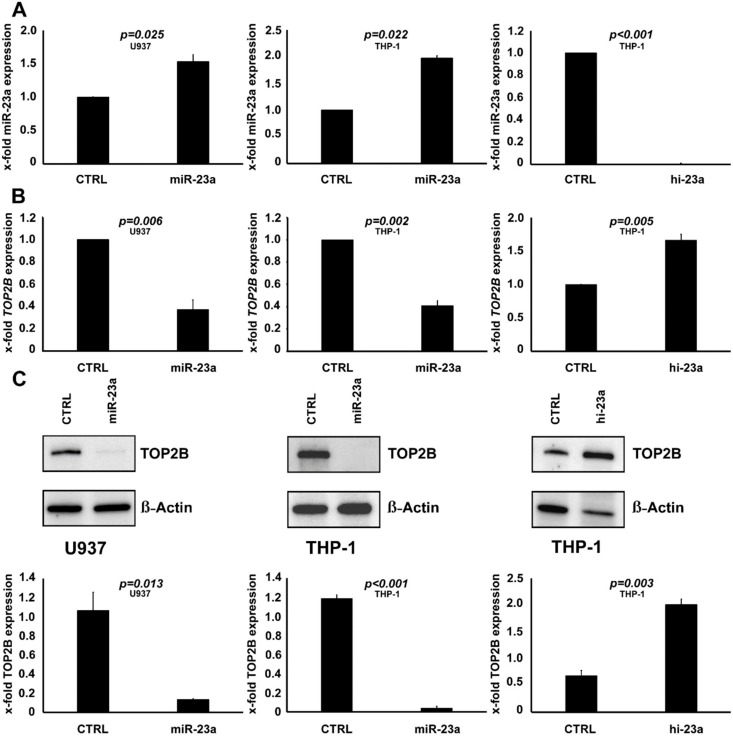
Modulation of *TOP2B* by miR-23a. (**A**) THP-1 and U937 cells were transfected with either miR-23a mimic (miR-23a) or unspecific control (CTRL). THP-1 were transfected with either miR-23a hairpin inhibitor (hi-23a) or control. miR-23a expression levels were analyzed by qPCR and are displayed as the x-fold expression of the control-transfected cells. (**B**) *TOP2B* mRNA expression analysis by qPCR of the conditions mentioned above demonstrated significant downregulation of *TOP2B* after transfection with miR-23a and significant upregulation after transfection with hi-23a. *TOP2B* expression is displayed as the x-fold expression of the control-transfected cells. (**C**) Immunoblot analyses demonstrating decreased expression of TOP2B protein in cells transfected with miR-23a and increased expression in conditions where hi-23a had been used. β-Actin served as the loading control. Below is the densitometric quantification of immunoblots showing the x-fold expression values of the control-transfected cells. Graphs demonstrate the mean of three independent experiments ± SD. Statistical significance was calculated using Student’s *t*-test.

We then sought to identify potential mechanisms behind miR-23a mediated chemoresistance in AML. We, therefore, performed in-silico screening for potential miR-23a target genes by employing the miR-walk 2.0 algorithm [36]. The TOP50 hits were subsequently subjected to a literature analysis, where we screened for a link to chemoresistance on the one hand, and to AML on the other hand (Supplementary Table S3). Only *TOP2B*, *ATP-binding cassette transporter 1* (*ABCA1*), and *monocyte-specific enhancer factor 2C* (*MEF2C*) fulfilled all of these criteria and were further evaluated. Next, we analyzed the expression of these genes within the TCGA-AML dataset [35], and correlated the results with the expression status of miR-23a. These analyses revealed a significant and inverse correlation between miR-23a and *TOP2B* only, which pinpoints a regulation of *TOP2B* by miR-23a (Supplementary Figure S3). To test this hypothesis, we analyzed *TOP2B* expression levels in the two AML cell lines with stable overexpression of miR-23a (THP-1 and U937). The results were further validated in THP-1 cells with a knockdown of miR-23a. In agreement with the clinical data, these experiments demonstrated that overexpression of miR-23a decreases the expression of TOP2B at mRNA and protein level, whereas, the knockdown of miR-23a has the opposite effect (Figure 4).

**Figure 5:**
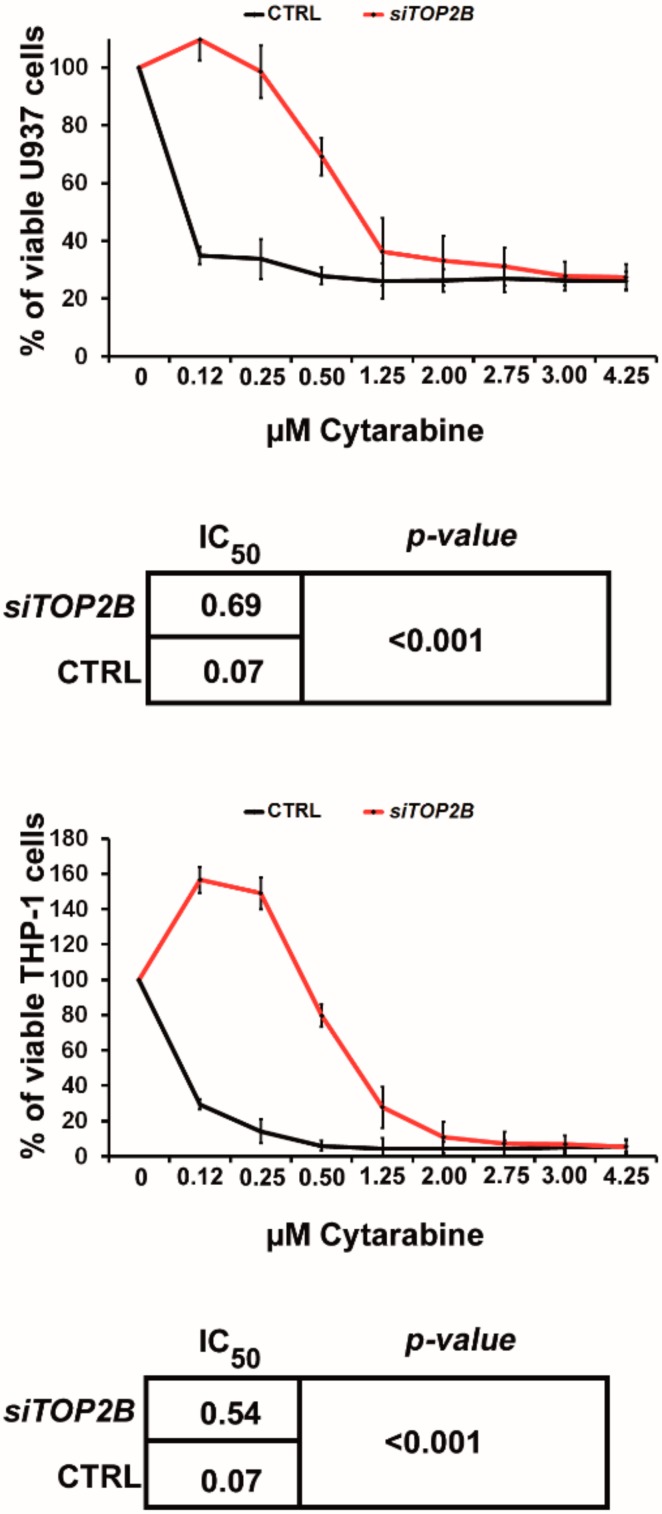
Sensitivity to cytarabine after *TOP2B* knockdown in AML cell lines. MTT cytotoxicity assays in AML cell lines after incubation with cytarabine. si*TOP2B* denotes transfection with a *TOP2B* siRNA; CTRL denotes transfection with a scrambled control siRNA. Experiments were repeated at least three times. The curves depict the mean ± SD. Statistical significance between IC_50_ values was calculated using Student’s *t*-test.

Having proven that miR-23a regulates *TOP2B*, we were interested in whether it plays a role in mediating resistance to AraC as well. As overexpression of miR-23a caused resistance to AraC and downregulation of *TOP2B*, we thereby hypothesized, that a knockdown of *TOP2B* should reduce the sensitivity to AraC as well. We, therefore, performed siRNA mediated knockdown of *TOP2B* in U937 and THP-1 cells. Then, we incubated these cells with increasing concentrations of AraC and performed MTT assays as outlined above. Indeed, knockdown of *TOP2B* decreased the sensitivity to AraC in both cell lines (Figure 5). Of note, despite the use of several expression constructs, we were not able to perform overexpression of *TOP2B* in any of the cell lines studied.

### 2.4. TOP2B Is a Direct Target of miR-23a

**Figure 6:**
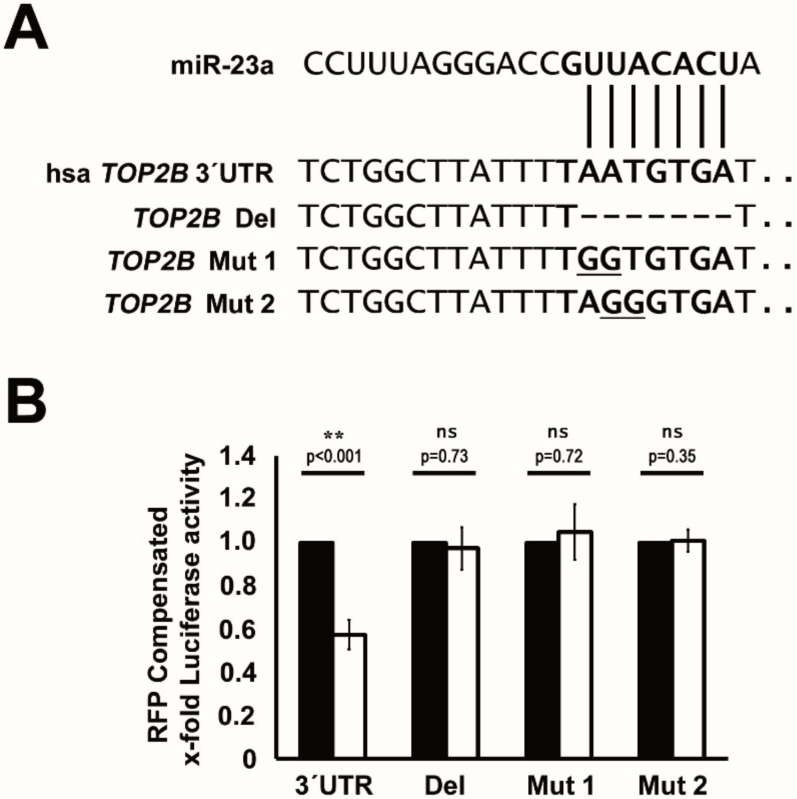
Modulation of *TOP2B* expression by direct binding of miR-23a in its 3′UTR. (**A**) The sequence of the human *TOP2B* 3′UTR showing the miR-23a–binding site. Matching base pairs are highlighted by lines. Alteration of the miR-23a binding site by either deletion (*TOP2B* Del) or two different mutations (*TOP2B* Mut 1 and Mut 2) is depicted. (**B**) HEK-293 cells were co-transfected with either miR-23a mimic (white bars) or unspecific control (CTRL, black bars), together with luciferase reporter clones, containing (i) wild-type *TOP2B* 3′UTR (3′UTR), (ii) *TOP2B* Del (Del), (iii) *TOP2B* Mut1 (Mut 1), or *TOP2B* Mut2 (Mut 2). Compensation for different transfection efficiencies was performed by co-transfection of pCS2-RFP. Graphs demonstrate the mean luciferase activity (RFP-compensated) of three independent experiments ± SD; values are given as the x-fold expression of the respective control-transfected setting. Statistical significance between IC_50_ values was calculated using Student’s *t*-test.

We next aimed to prove that miR-23a-mediated regulation of *TOP2B* is caused by direct binding to its 3′UTR. Initially, we screened the *TOP2B* 3′UTR in more detail and identified one potential binding site for miR-23a (Figure 6A). In the next step, we employed an expression construct, where the 3′UTR of *TOP2B* was attached to the coding region of luciferase. Importantly, co-transfection of this construct with a miR-23a mimic caused a significant downregulation of luciferase activity, whereas, scrambled miR-controls failed to do so (Figure 6B). To further prove that miR-23a regulates *TOP2B* by direct interaction, we then altered the 3′UTR of *TOP2B* by introducing two different mutations and deletion, respectively, in the putative binding site. Indeed, this caused an inhibition of the miR-23a–mediated downregulation of luciferase activity (Figure 6B), which indicates that miR-23a regulates *TOP2B* expression by direct interaction within its 3′UTR.

### 2.5. Decreased Expression of TOP2B Correlates with Relapsed/Refractory AML and with Shorter Survival in AraC-Treated Patients

**Figure 7:**
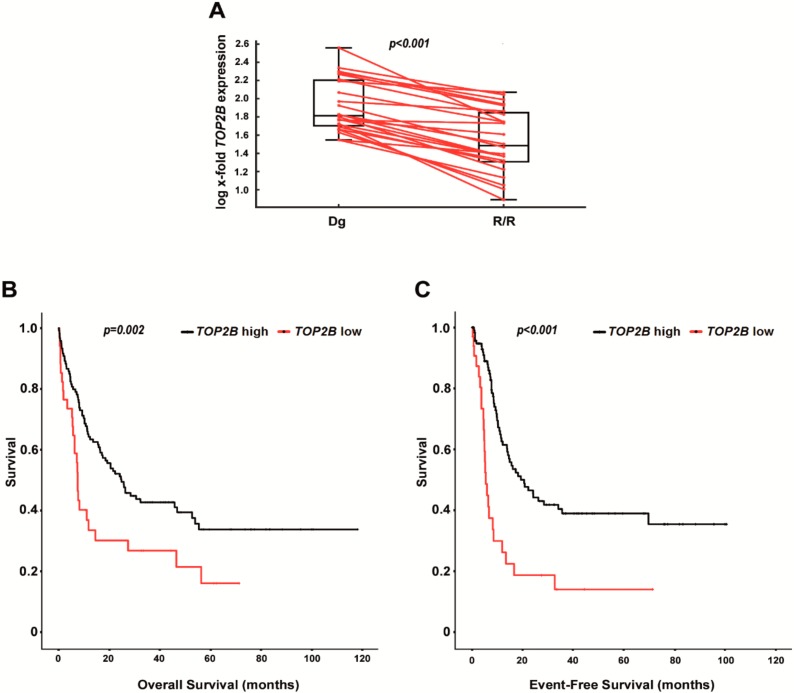
Expression of *TOP2B* in primary AML patient specimens. (**A**) Box plots displaying *TOP2B* mRNA expression levels in 24 paired AML patient specimens collected at the stage of diagnosis (Dg) and relapsed/refractory disease (R/R). miR-23a expression levels were analyzed by qPCR and are displayed as the log-transformed x-fold expression of the calibrator (NB4 cells). The *p*-value has been calculated using the Wilcoxon signed-rank test. (**B**,**C**) Overall survival (**B**) and event-free survival (**C**) in AML patients treated with AraC-containing high-dose regimens (n = 154) according to the *TOP2B* expression status. Data were downloaded from the TCGA-AML dataset [35], and re-analyzed for the expression of *TOP2B*. Statistical significance was calculated by Cox regression analysis.

Finally, we aimed to delineate the clinical relevance of *TOP2B* downregulation in AraC-based AML therapy. As outlined above for miR-23a, we initially analyzed the expression of *TOP2B* in the 24 paired AML patient specimens. Again, we compared *TOP2B* expression levels measured at diagnosis, with the ones assessed at R/R stages. Contrary to miR-23a, *TOP2B* expression was significantly decreased at the stage of R/R disease (Figure 7A), which is in agreement with the in-vitro data showing that *TOP2B* knockdown reduces the sensitivity to AraC. To validate these data in an independent cohort, we again studied *TOP2B* expression within the TCGA-AML dataset [35], and correlated the results with survival data in patients treated with AraC-containing high-dose regimens (*n* = 154). Indeed, lower *TOP2B* expression levels associated with both shorter EFS and OS (Figure 7B), which again supports the role of *TOP2B* downregulation in the development of AraC chemoresistance in AML. Again, we aimed for a multivariate model with the inclusion of the established AML risk factors age at diagnosis, WBC and cytogenetics. As seen for miR-23a, *TOP2B* expression thereby remained statistically significant, which proves its independent value as a predictive biomarker in AraC-treated AML (Table 2).

Finally, we aimed to explore whether the relevance of the miR-23a/*TOP2B* axis in predicting survival in AraC-treated patients might also be depicted in the analysis of a more generalized gene signature, as previously shown for LSCs and genes associated with high oxidative phosphorylation (OXPHOS) status [37,38]. As described above, we employed the miR-walk 2.0 algorithm [36], and performed an in-silico analysis for potential miR-23a target genes. The TOP300 hits were subsequently correlated with miR-23a expression within the TCGA dataset. This revealed a list of genes with a statistically significant downregulation in patients with high miR-23a expression (miR-23a DOWN; *n* = 32 genes), and a list of 15 genes with increased expression in this patient cohort (miR-23a UP; Supplementary Table S4). Additionally, we aimed to test a putative *TOP2B* signature, which comprised a list of previously described *TOP2B* interaction partners (*TOP2B* Interact; *n* = 25 genes; Supplementary Table S4) [39]. We then aimed to elaborate on the performance of these signatures in predicting the survival of AraC-treated patients. In univariate analyses, miR-23a DOWN correlated with OS and EFS. However, this significance got lost in multivariate models. All other signatures were not associated with any of the survival parameters tested (Supplementary Table S5). Additionally, we did not observe an overlap with the previously presented LSC and OXPHOS signatures [37,38], respectively (Supplementary Table S4). Hence, our data suggest that the miR-23a/*TOP2B* axis represents a specific modulator of AraC sensitivity rather than being part of a more generalized drug resistance profile.

## 3. Discussion

Chemoresistance is one of the significant obstacles in the therapy of AML. Identification of molecular targets that mediate chemoresistance, and to use them for the establishment of novel treatment approaches has become a central field of AML research with a tremendous potential to increase survival if successful. A constantly growing list of candidate genes has already been described. Lately, miRNAs have been identified as novel players within the development of chemorefractory disease [5]. In this study, we analyzed the role of miR-23a in chemoresistance to AraC and daunorubicin, which form the backbone of the classical 7 + 3 induction scheme in AML. Thereby, we discovered, that increased expression of miR-23a reduces the sensitivity to AraC in three different AML cell lines and correlates with inferior treatment outcomes in primary AML patient specimens. Although other groups have described a functional role of miR-23a in the pathogenesis of AML and us previously [26,27,28], its role in the development of AML chemoresistance is novel. These results are in agreement with data from solid tumors, where miR-23a conferred resistance to cisplatin and 5-fluorouracil [29,30,31,32], and thereby establish miR-23a as a global player in the development of chemotherapeutic resistance. In this respect, it is also necessary to mention that our study has limitations. These include the fact that the selection of cell lines cannot cover the whole array of genetic aberrations described in AML, and that the clinical patient data were obtained from a retrospectively collected dataset. Future studies, which delineate the role of miR-23a in AraC resistance in in-vitro systems with a more stringent molecular profile, in in-vivo preclinical models, as well as in specifically designed, prospective patient cohorts, are therefore, warranted.

An interesting aspect of our study is the observation that miR-23a expression is increased at R/R stages of AML. These data suggest the existence of two possibilities for miR-23a mediated chemoresistance in AML patients: (i) Cells with high miR-23a expression display one of the major clones already at diagnosis, which results in a primary chemorefractory situation; (ii) high miR-23a expression is initially only present in the small fraction of LSCs. In this well-known concept, the bulk AML is successfully cleared by chemotherapy [12,13,14,15,16]. However, a chemoresistant LSC compartment remains. These LSCs then establish the AML relapse, which shares the molecular profile with the parental LSC pool, and therefore, exhibits chemoresistance. Indeed, our re-analysis of a previously published miRNA array expression profiling in functionally validated LSCs and corresponding AML bulk specimens [13] pinpoint increased expression of miR-23a in the LSC pool, and therefore, suggest, that miR-23a might be an LSC marker which helps the LSC compartment in achieving chemoresistance to AraC. These results are also in agreement with data from solid cancers, where high miR-23a expression correlated to the cancer stem cell (CSC) pool as well. Jahid and coworkers demonstrated that miR-23a is amongst the most highly expressed miRNAs in colorectal CSCs [40]. They also show a functional relevance of these findings, as miR-23a promoted the migration and invasion of the colorectal CSC pool, thereby contributing to the formation of metastases. Han and coworkers reported an increased expression of miR-23a in CSC of non-small cell lung cancer (NSCLC), as compared to the corresponding non-CSC tumor cells [41]. Moreover, the isolated CSCs were particularly resistant to treatment with the epidermal growth factor receptor inhibitor erlotinib. These data are interesting, as it is nowadays believed that targeted therapies can only cure a patient if they manage to eradicate cancer/leukemic stem cell pool. In this respect, the authors demonstrated that miR-23a depletion re-sensitized NSCLC-CSC to erlotinib and thereby present a feasible possibility for a novel and CSC-directed therapeutic approach in NSCLC. Whether miR-23a provides a target to sensitize LSCs for cytotoxic agents used in AML is currently unclear and remains to be determined in future studies, specifically addressing this issue.

While the concept of miR-23a as a putative LSC marker is appealing, it is also worth to mention that miR-23a might additionally mediate its effects on AraC resistance via other, LSC-independent biologic functions. In a recent publication, Farge and colleagues presented data suggesting that AraC resistance in AML is not primarily mediated via LSC enrichment, but via the enrichment of cells with a high OXPHOS status [37]. They present a specific OXPHOS gene signature, which is predictive for the treatment response in both preclinical AML models and patients with AML. To screen for a potential link with miR-23a expression, we aimed to established a miR-23a gene signature. This list contained genes, which were (i) predicted as miR-23a target genes in an in-silico analysis, and (ii) which additionally correlated with miR-23a expression within the TCGA AML dataset. We thereby failed to detect a significant overlap between the miR-23a and OXPHOS gene signatures, which suggest that the miR-23a/*TOP2B* axis represents a specific modulator of AraC sensitivity rather than being part of a more generalized drug resistance profile. Another possible mechanism of miR-23a-mediated AraC resistance would be modulation of the bone marrow microenvironment. The composition of the tumor microenvironment has been shown to be fundamental for the growth and survival of tumors, and lately, a role in mediating resistance to cytotoxic agents has been established as well [8,9,10,11]. miR-23a has been demonstrated to modulate the microenvironment of several tumors previously [42]. Hence, one might speculate that parts of the effects on AraC resistance might be mediated rather via changes in the microenvironment than via increased expression within the leukemia cells only. These hypotheses cannot be verified with in-vitro experiments only. Hence, it will be essential to design in-vivo models, specifically addressing these questions in the near future.

In addition to its attractiveness as a therapeutic target, miR-23a might also serve as a potential biomarker. In more detail, high miR-23a expression might help to select patients, in whom a suboptimal response to AraC-based therapies has to be expected. This knowledge would be particularly relevant for older patients, where the use of high-dose induction therapy is still a matter of debate, and where alternative low-intensity regimens, such as low-dose AraC and hypomethylating agents, exist [4,43,44,45]. Such use of miR-23a as a biomarker is further fueled by the fact that miRNAs were successfully established as biomarkers previously and that they offer several advantages over conventional gene mRNAs [32,46]. In particular, their small size provides protection during tissue processing, thereby guaranteeing stability and consequently reproducibility of the expression analyses. In the hematologic system, expression analysis of various miRNAs is possible from both, buffy coats and formalin-fixed paraffin-embedded tissues, the latter including decalcified bone marrow biopsies [28,46]. One might hypothesize that in such a scenario, miR-23a expression profiling might help to identify patients who profit from non-AraC-based low-intensity therapies. However, it has to be noted that it is currently unknown whether miR-23a affects the sensitivity to these agents as well. Future laboratory and clinical studies will be warranted to test this hypothesis.

Searching for mechanisms behind miR-23a mediated AraC resistance, we initially screened for potential miR-23a target genes. By focusing on candidates that were associated with chemoresistance and AML previously, we identified *TOP2B* as a direct target of miR-23a. We were able to demonstrate that miR-23a binds the 3′UTR of *TOP2B* and thereby downregulates its expression, both at the mRNA and protein level. Topoisomerases are essential enzymes in DNA replication and transcription, as they help to resolve topological problems arising during these processes [47,48]. They introduce single- and double-strand breaks, respectively, thereby enabling each DNA strand to be correctly positioned, and finally catalyze the correct religation of both strands [48]. Topoisomerase-inhibitors and anthracyclines trap the topoisomerase-induced process after the creation of DNA strand breaks. Thereby, they prevent resealing of the DNA, which in turn induces apoptosis of the affected cells. Reduced expression of topoisomerases can mediate resistance to topoisomerase inhibitors and/or anthracyclines, as a reduction of topoisomerase enzymes results in a decreased number of cleaved DNA strands that can be trapped [47,48]. Surprisingly, however, our functional in-vitro assays revealed that knockdown of *TOP2B* reduced the sensitivity to AraC, a nucleoside analog and antimetabolite. It thereby mimics the effects of miR-23a overexpression, which suggests that the downregulation of *TOP2B* might execute the miR-23a-mediated AraC resistance. While these findings were unexpected, the reduction of AraC sensitivity by decreased topoisomerase expression levels has been reported previously [49]. Pourquier and colleagues demonstrated that AraC incorporation alters the structure of DNA and that its incorporation immediately 3′ of a DNA topoisomerase 1 (*TOP1*) cleavage site enhanced the formation of *TOP1*-induced single-strand breaks. Mechanistically, AraC incorporation prevented the *TOP1*-mediated DNA religation, which suggests that topoisomerase inhibition contributes to the anti-tumoral effects of AraC as well. In line with these data and with the data presented in the current study, the authors reported that *TOP1* deficient cells were resistant to the treatment with AraC. Our data are also in agreement with a study from Song and colleagues, who studied the expression of *TOP2A* and *TOP2B* in 54 primary, 7 + 3 treated AML patient specimens [50]. Increased *TOP2B* levels correlated with a prolonged survival within this cohort. Of note, miR-23a overexpression failed to mediate resistance to daunorubicin in our experiments. In this respect, it has to be noted that every miR regulates the expression of a large number of target genes, which are only known to a certain extent at this time [17,18]. Moreover, the extent of target gene regulation has also been shown to be tissue-specific [5]. As a consequence, miR-23a overexpression in AML cells will most likely induce specific deregulation of gene expression profiles, which will not only include the down-regulation of *TOP2B* but also the up- and/or downregulation of many other genes. One might hypothesize that the failure of miR-23a overexpression to induce daunorubicin resistance might be caused by the deregulation of other, hitherto unknown effector genes, which specifically protect miR-23a overexpressing leukemia cells from daunorubicin-resistance.

## 4. Materials and Methods

### 4.1. Patient Samples and Cell Lines

Primary AML patient specimens were collected at the stage of diagnosis and the stage of relapse or primary chemorefractory disease (R/R stage) at the Division of Hematology, Medical University of Graz, Graz, Austria (MUG). All samples were processed by Ficoll density gradient centrifugation, and mononuclear cell fractions were stored in the Biobank at the MUG until further use as described previously [51,52,53,54,55,56]. Additionally, cytospin preparations from mononuclear cell layers were analyzed, and only samples with a blast cell percentage of >80% were retained for further analyses. Classification of AML was performed according to the World Health Organization (WHO) guidelines [57]. AML cell lines (THP-1, U937, HL-60) were purchased from the German Collection of Microorganisms and Cell Cultures (Braunschweig, Germany). HEK-293 and 293-T cells were obtained from the Core Facility Alternative Biomodels and Preclinical Imaging (Center for Medical Research at the MUG). Stocks from low passages were frozen, and cells always were kept in culture for less than six months after thawing. Cells were authenticated regularly by a variable number of tandem repeat DNA profiling (VNTR) as described earlier [28,51,58,59]. The study was approved by the institutional review board of the Medical University of Graz (EK 30-464 ex 17/18), and informed consent was obtained from all individuals.

### 4.2. Cell Culture, Lentiviral Transduction and Transfection

Cells were cultivated at 37 °C/5% CO_2_ in RPMI-1640 (THP-1, U937 and HL-60) and DMEM (HEK-293 and 293-T), respectively (all from Sigma-Aldrich, St. Louis, MO, USA). All media were supplemented with 10% heat-inactivated FCS and 1× antibiotic-antimycotic, including 100 μg/mL streptomycin, 100 U/mL penicillin, and 0.25 μg/mL amphotericin B (Thermo Fisher Scientific, Waltham, MA, USA). Lentiviral transduction for overexpression of miR-23a-3p in THP-1 and U937 was performed using a psi-pEZX-MR03 expression construct (Genecopeia, Rockville, MD, USA) as previously described [28,51,53]. Stable selection and maintenance were performed in the presence of 1.0 μg/mL puromycin.

For transient transfection, miR-23a-3p hairpin inhibitors and miR-23a-3p mimics (Dharmacon, Lafayette, CO, USA and Qiagen, Hilden, Germany), as well as *TOP2B siRNA* (ON-TARGETplus *TOP2B* siRNA, Dharmacon) were transfected at a concentration of 20 nmol/L using DharmaFECT2 (Dharmacon) for THP-1, U937 and HL-60 or Lipofectamine RNAiMAX (Thermo Fisher Scientific) for adherent cell lines. Transfections were always performed with the respective scrambled controls. Chemoresistance assays were performed 48 h after transfection. For luciferase reporter assays, HEK-293 were co-transfected with 0.5 ng/μL pCS2-red fluorescent protein (RFP) and 0.25 ng/μL *TOB2B*-3′-untranslated region (UTR)-pMirTarget (all from Origene, Rockville, MA, USA), and miR-23a-3p mimics or scrambled control (both at a concentration of 80 nmol/L). The RFP-construct was included to enable compensation of differences in transfection efficiency.

### 4.3. qPCR and Immunoblot Analysis

Whole RNA was extracted from patient samples and cell lines using TRIzol® (Invitrogen, Carlsbad, CA, USA) according to the manufacturer’s protocol. cDNA was synthesized from 1 µg RNA using the Taq Man® Reverse Transcription Reagents (Applied Biosystems, Foster City, CA, USA) for mRNA and miScript II Reverse Transcriptase Kit (Qiagen) for miRNA, respectively. Real-time quantitative PCR (qPCR) was performed using the ΔΔ*C*_t_ method, as previously described [51,53,59,60,61,62]. *B2M* and *GUSB* were employed as control genes for mRNA analyses, whereas, *RNU6* and *SNORD44* were used for miRNA expression profiling. Primer sequences are displayed in Supplementary Table S6. NB4 (for miRNA qPCR of the patient samples) and cells transfected with scrambled controls (for all other experiments), respectively, served as calibrators. Immunoblot was performed as described previously [51,53,59,61,62], using the following antibodies: Anti-TOP2B (#sc-365071; Santa Cruz, Dallas, TX, USA) and anti-β-Actin (#A5441; Sigma Aldrich, St. Louis, MO, USA).

### 4.4. Chemosensitivity Assays

For MTT assays, 3 × 10^4^ cells were seeded in 96-well plates and then treated with cytarabine (Sigma-Aldrich) and daunorubicin (Sigma-Aldrich), respectively. Both substances were dissolved in RPMI-1640 supplemented with 10% FBS. After a 48 h incubation period, the CellTiter 96 Non-Radioactive Cell Proliferation Assay (Promega, Madison, WI, USA) was performed according to the manufacturer’s protocol. Briefly, 20 µL MTT assay solution was added into each well for 4 h, followed by 10-min centrifugation at 2500 rpm to allow for sedimentation of the purple-colored precipitate of formazan. After discarding the supernatant, the precipitate was dissolved in 200 µL DMSO, and the absorbance was detected at 490 nm wavelength using an automatic multi-well spectrophotometer InfiniteF50 (Tecan, Männedorf, Switzerland). For clonogenic assays, cells were pre-incubated with 5 µM cytarabine and empty dissolvant, respectively, for 2 h in 48-well plates. Soft-agar assays were set up in 6-well plates, each well containing 2 mL of a bottom layer of 0.5% SeaPlaque agarose (Biozym Scientific GmbH, Hessisch Oldendorf, Germany) and a top layer (2 mL) of 0.4% agarose containing 7500 cells. The plates were incubated for 8–10 days. To assess colony frequencies, at least 15 pictures were taken per well, and large colonies (>5000 pixels) were quantified with ImageJ (https://imagej.net). Ratios of colonies in AraC-treated conditions to the respective untreated controls were calculated and compared.

### 4.5. Luciferase Reporter Assays

Luciferase reporter assays were performed 24 h after transfection as previously described [28]. In more detail, luciferase activity and RFP fluorescence values were determined to utilize a Cytation 5 Plate reader (BioTek, Winooski, VT, USA). Thereby, 100 μL of luciferase Assay System Reagent (Promega, Madison, WI, USA) were injected automatically into each well of a 96 well plate containing 20 μL of cell lysate, the provided cell culture lysing reagent was used as background control. Luminescence was measured for 10 s after a delay of 2 s. Fluorescence was detected with a 544/616 nm filter set.

### 4.6. Database Retrieval and Statistical Analyses

Expression data for *TOP2B* (obtained by RNA Sequencing V2 RSEM) and miRNAs (obtained by miRNA Sequencing) from de-novo AML patients treated with high-dose chemotherapy containing AraC were downloaded and analyzed from the AML dataset [35] of The Cancer Genome Atlas (TCGA, http//:www.cancergenome.nih.gov) on 15 January 2018. In case of availability, data were downloaded and analyzed using the cBioPortal for Cancer Genomics (http://www.cbioportal.org/public-portal/index.do) [63,64]. For LSC analyses, previously published miR-microarray expression data [13] were downloaded from the Gene Expression Omnibus (http://www.ncbi.nlm.nih.gov/geo; accession number GSE55916; download 19 August 2019) and re-analyzed for the expression of miR-23a.

Wilcoxon signed-rank tests were employed for comparison of miR-23a, as well as *TOP2B* expression levels between primary patient samples at diagnosis and R/R stages, as well as for comparison of miR-23a expression between LSCs and corresponding AML bulk samples. Correlation coefficients between miR-23a and gene expression levels were calculated by Spearman-Rho. Effects of miR-23a and *TOP2B* expression on event-free survival (EFS) and overall survival (OS), respectively, were calculated by Cox regression analysis. Additionally, miR-23a and *TOP2B* expression levels were categorized as dichotomous variables (samples with low versus samples with high expression). The optimal cut-off to separate these groups was assessed by employing a maximized Youden’s Index within a receiver operating characteristic (ROC) analysis [65] of EFS and subsequently applied for EFS and OS. Associations with OS and EFS, respectively, were calculated in both uni- and multivariable Cox proportional hazards regression models. Beside miR-23a and *TOP2B*, respectively, these calculations included the well-established AML risk factors age, white blood cell count (WBC), and cytogenetic risk group.

For analysis of in vitro experiments, Student’s *t*-test was calculated from at least three independent experiments. Half maximal inhibitory concentration (IC_50_) values were calulated from a 4-parameter logistic dose-response model. Differences in mean IC_50_ between treatments and controls were tested by Student’s *t*-test. All analyses were performed using SPSS 22.0 (SPSS Inc., Chicago, IL, USA) and R 3.6.1 (http//:www.r-project.org), respectively. All statistical tests were performed two-sided, and a *p*-value of <0.05 was considered statistically significant.

## 5. Conclusions

In conclusion, we demonstrate that increased expression of miR-23a in AML mediates resistance to AraC and correlates with an inferior outcome in AraC-treated AML patients. We also show that miR-23a expression is particularly high at the stage of the primary chemoresistant disease and/or chemoresistant relapse and that it is linked to the intrinsically resistant LSC pool. Mechanistically, we demonstrate that miR-23a causes the downregulation of *TOP2B*, which is likely to mediate its effects on AraC sensitivity.

## Figures and Tables

**Table 1:** Multivariate Cox regression analysis for EFS and OS. Statistically significant values are indicated in bold. EFS, event-free survival; OS, overall survival; CI, confidence interval; WBC, white blood cells; G/L, giga per litre.

miR-23a				
Parameter	Variable	Hazard Ratio	95% CI	*p*-Value
OS	miR-23a high	2.862	1.385–5.913	**0.005**
	WBC, G/L	1.000	1.000–1.001	0.637
	Age at diagnosis	1.019	1.002–1.035	**0.024**
	Cytogenetics	1.418	1.013–1.984	**0.042**
EFS	miR-23a high	1.854	1.023–3.363	**0.042**
	WBC, G/L	1.000	1.000–1.001	0.279
	Age at diagnosis	1.011	0.996–1.025	0.144
	Cytogenetics	1.549	1.130–2.110	**0.006**

**Table 2:** Multivariate Cox regression analysis for EFS and OS. Statistically significant values are indicated in bold. EFS, event-free survival; OS, overall survival; CI, confidence interval; WBC, white blood cells; G/L, giga per litre.

*TOP2B*				
Parameter	Variable	Hazard Ratio	95% CI	*p*-Value
OS	TOP2B high	0.581	0.367–0.922	**0.021**
	WBC, G/L	1.000	1.000–1.001	0.092
	Age at diagnosis	1.031	1.015–1.047	**<0.001**
	Cytogenetics	1.844	1.328–2.561	**<0.001**
EFS	TOP2B high	0.399	0.245–0.650	**<0.001**
	WBC, G/L	1.000	1.000–1.001	0.067
	Age at diagnosis	1.009	0.994–1.025	0.232
	Cytogenetics	1.513	1.056–2.168	**0.024**

## Supplementary Materials

The following are available online at https://www.mdpi.com/2072-6694/12/2/496/s1, Figure S1: Sensitivity to daunorubicin after miR–23a modulation in AML cell lines, Figure S2: Expression of miR–23a does not correlate with NPM1 and/or FLT3 mutation status in AML, Figure S3: Increased expression of miR–23a correlates with decreased expression of TOP2B in AML, Figure S4. Uncropped Western Blots, Table S1: Patient characteristics of the 24 paired AML specimens obtained at diagnosis and primary chemorefractory/relapsed disease, Table S2: Patient characteristics of the 146 AML patients analyzed via the TCGA [1], Table 3: TOP50 hits of the in–silico screening for potential miR–23a target genes by employing the miR–walk 2.0 algorithm ([2]; http://zmf.umm.uni–heidelberg.de/apps/zmf/mirwalk2/). Table S4: Gene signatures of miR–23a and TOP2B in comparison to the previously published LSC and OXPHOS signatures [3,4], Table S5. Univariate Cox regression analysis for EFS and OS for the miR–23a and TOP2B gene signatures.

## References

[1] Dohner H., Weisdorf D.J., Bloomfield C.D. (2015). Acute Myeloid Leukemia. N. Engl. J. Med..

[2] Koreth J., Schlenk R., Kopecky K.J., Honda S., Sierra J., Djulbegovic B.J., Wadleigh M., DeAngelo D.J., Stone R.M., Sakamaki H. (2009). Allogeneic Stem Cell Transplantation for Acute Myeloid Leukemia in First Complete Remission: Systematic Review and Meta-Analysis of Prospective Clinical Trials. JAMA.

[3] Magina K.N., Pregartner G., Zebisch A., Wolfler A., Neumeister P., Greinix H.T., Berghold A., Sill H. (2017). Cytarabine Dose in the Consolidation Treatment of AML: A Systematic Review and Meta-Analysis. Blood.

[4] Dohner H., Estey E., Grimwade D., Amadori S., Appelbaum F.R., Buchner T., Dombret H., Ebert B.L., Fenaux P., Larson R.A. (2017). Diagnosis and Management of AML in Adults: 2017 ELN Recommendations from an International Expert Panel. Blood.

[5] Zebisch A., Hatzl S., Pichler M., Wolfler A., Sill H. (2016). Therapeutic Resistance in Acute Myeloid Leukemia: The Role of Non-Coding RNAs. Int. J. Mol. Sci..

[6] Juliusson G., Lazarevic V., Horstedt A.S., Hagberg O., Hoglund M., Swedish Acute Leukemia Registry Group (2012). Acute Myeloid Leukemia in the Real World: Why Population-Based Registries are Needed. Blood.

[7] Juliusson G., Antunovic P., Derolf A., Lehmann S., Mollgard L., Stockelberg D., Tidefelt U., Wahlin A., Hoglund M. (2009). Age and Acute Myeloid Leukemia: Real World Data on Decision to Treat and Outcomes from the Swedish Acute Leukemia Registry. Blood.

[8] Bakker E., Qattan M., Mutti L., Demonacos C., Krstic-Demonacos M. (2016). The Role of Microenvironment and Immunity in Drug Response in Leukemia. Biochim. Biophys. Acta.

[9] Rashidi A., Uy G.L. (2015). Targeting the Microenvironment in Acute Myeloid Leukemia. Curr. Hematol. Malign Rep..

[10] Tabe Y., Konopleva M. (2015). Role of Microenvironment in Resistance to Therapy in AML. Curr. Hematol. Malign Rep..

[11] Vasan N., Baselga J., Hyman D.M. (2019). A View on Drug Resistance in Cancer. Nature.

[12] Kreso A., Dick J.E. (2014). Evolution of the Cancer Stem Cell Model. Cell Stem Cell.

[13] Lechman E.R., Gentner B., Ng S.W., Schoof E.M., van Galen P., Kennedy J.A., Nucera S., Ciceri F., Kaufmann K.B., Takayama N. (2016). MiR-126 Regulates Distinct Self-Renewal Outcomes in Normal and Malignant Hematopoietic Stem Cells. Cancer Cell.

[14] Saito Y., Kitamura H., Hijikata A., Tomizawa-Murasawa M., Tanaka S., Takagi S., Uchida N., Suzuki N., Sone A., Najima Y. (2010). Identification of Therapeutic Targets for Quiescent, Chemotherapy-Resistant Human Leukemia Stem Cells. Sci. Transl. Med..

[15] Thomas D., Majeti R. (2017). Biology and Relevance of Human Acute Myeloid Leukemia Stem Cells. Blood.

[16] Reinisch A., Chan S.M., Thomas D., Majeti R. (2015). Biology and Clinical Relevance of Acute Myeloid Leukemia Stem Cells. Semin. Hematol..

[17] Ling H., Fabbri M., Calin G.A. (2013). MicroRNAs and Other Non-Coding RNAs as Targets for Anticancer Drug Development. Nat. Rev. Drug Discov..

[18] Pichler M., Calin G.A. (2015). MicroRNAs in Cancer: From Developmental Genes in Worms to their Clinical Application in Patients. Br. J. Cancer.

[19] Zebisch A., Caraffini V., Sill H. (2019). RAF Kinase Inhibitor Protein in Myeloid Leukemogenesis. Int. J. Mol. Sci..

[20] Barth D.A., Slaby O., Klec C., Juracek J., Drula R., Calin G.A., Pichler M. (2019). Current Concepts of Non-Coding RNAs in the Pathogenesis of Non-Clear Cell Renal Cell Carcinoma. Cancers.

[21] Klec C., Prinz F., Pichler M. (2019). Involvement of the Long Noncoding RNA NEAT1 in Carcinogenesis. Mol. Oncol..

[22] Smolle M.A., Prinz F., Calin G.A., Pichler M. (2019). Current Concepts of Non-Coding RNA Regulation of Immune Checkpoints in Cancer. Mol. Asp. Med..

[23] Ling H., Vincent K., Pichler M., Fodde R., Berindan-Neagoe I., Slack F.J., Calin G.A. (2015). Junk DNA and the Long Non-Coding RNA Twist in Cancer Genetics. Oncogene.

[24] Zhang X.W., Liu N., Chen S., Wang Y.E., Sun K.L., Xu Z.M., Fu W.N. (2015). Upregulation of microRNA-23a Regulates Proliferation and Apoptosis by Targeting in Laryngeal Carcinoma. Oncol. Lett..

[25] Chhabra R., Dubey R., Saini N. (2010). Cooperative and Individualistic Functions of the microRNAs in the miR-23a~27a~24-2 Cluster and its Implication in Human Diseases. Mol. Cancer.

[26] Zhang Y.C., Ye H., Zeng Z., Chin Y.E., Huang Y.N., Fu G.H. (2015). The NF-kappaB p65/miR-23a-27a-24 Cluster is a Target for Leukemia Treatment. Oncotarget.

[27] Zhao C., Wang S., Zhao Y., Du F., Wang W., Lv P., Qi L. (2019). Long Noncoding RNA NEAT1 Modulates Cell Proliferation and Apoptosis by Regulating miR-23a-3p/SMC1A in Acute Myeloid Leukemia. J. Cell. Physiol..

[28] Hatzl S., Geiger O., Kuepper M.K., Caraffini V., Seime T., Furlan T., Nussbaumer E., Wieser R., Pichler M., Scheideler M. (2016). Increased Expression of miR-23a Mediates a Loss of Expression in the RAF Kinase Inhibitor Protein RKIP. Cancer Res..

[29] Li X., Li X., Liao D., Wang X., Wu Z., Nie J., Bai M., Fu X., Mei Q., Han W. (2015). Elevated microRNA-23a Expression Enhances the Chemoresistance of Colorectal Cancer Cells with Microsatellite Instability to 5-Fluorouracil by Directly Targeting ABCF1. Curr. Protein Pept. Sci..

[30] Peng F., Zhang H., Du Y., Tan P. (2015). MiR-23a Promotes Cisplatin Chemoresistance and Protects Against Cisplatin-Induced Apoptosis in Tongue Squamous Cell Carcinoma Cells through Twist. Oncol. Rep..

[31] Yu Z.W., Zhong L.P., Ji T., Zhang P., Chen W.T., Zhang C.P. (2010). MicroRNAs Contribute to the Chemoresistance of Cisplatin in Tongue Squamous Cell Carcinoma Lines. Oral Oncol..

[32] Komatsu S., Ichikawa D., Kawaguchi T., Takeshita H., Miyamae M., Ohashi T., Okajima W., Imamura T., Kiuchi J., Arita T. (2016). Plasma microRNA Profiles: Identification of miR-23a as a Novel Biomarker for Chemoresistance in Esophageal Squamous Cell Carcinoma. Oncotarget.

[33] Fleming R.A., Capizzi R.L., Rosner G.L., Oliver L.K., Smith S.J., Schiffer C.A., Silver R.T., Peterson B.A., Weiss R.B., Omura G.A. (1995). Clinical Pharmacology of Cytarabine in Patients with Acute Myeloid Leukemia: A Cancer and Leukemia Group B Study. Cancer Chemother. Pharmacol..

[34] Valent P., Sadovnik I., Eisenwort G., Bauer K., Herrmann H., Gleixner K.V., Schulenburg A., Rabitsch W., Sperr W.R., Wolf D. (2019). Immunotherapy-Based Targeting and Elimination of Leukemic Stem Cells in AML and CML. Int. J. Mol. Sci..

[35] Cancer Genome Atlas Research Network (2013). Genomic and Epigenomic Landscapes of Adult De Novo Acute Myeloid Leukemia. N. Engl. J. Med..

[36] Dweep H., Gretz N. (2015). MiRWalk2.0: A Comprehensive Atlas of microRNA-Target Interactions. Nat. Methods.

[37] Farge T., Saland E., de Toni F., Aroua N., Hosseini M., Perry R., Bosc C., Sugita M., Stuani L., Fraisse M. (2017). Chemotherapy-Resistant Human Acute Myeloid Leukemia Cells are Not Enriched for Leukemic Stem Cells but Require Oxidative Metabolism. Cancer Discov..

[38] Eppert K., Takenaka K., Lechman E.R., Waldron L., Nilsson B., van Galen P., Metzeler K.H., Poeppl A., Ling V., Beyene J. (2011). Stem Cell Gene Expression Programs Influence Clinical Outcome in Human Leukemia. Nat. Med..

[39] Uuskula-Reimand L., Hou H., Samavarchi-Tehrani P., Rudan M.V., Liang M., Medina-Rivera A., Mohammed H., Schmidt D., Schwalie P., Young E.J. (2016). Topoisomerase II Beta Interacts with Cohesin and CTCF at Topological Domain Borders. Genome Biol..

[40] Jahid S., Sun J., Edwards R.A., Dizon D., Panarelli N.C., Milsom J.W., Sikandar S.S., Gumus Z.H., Lipkin S.M. (2012). MiR-23a Promotes the Transition from Indolent to Invasive Colorectal Cancer. Cancer Discov..

[41] Han Z., Zhou X., Li S., Qin Y., Chen Y., Liu H. (2017). Inhibition of miR-23a Increases the Sensitivity of Lung Cancer Stem Cells to Erlotinib through PTEN/PI3K/Akt Pathway. Oncol. Rep..

[42] Wang N., Tan H.Y., Feng Y.G., Zhang C., Chen F., Feng Y. (2018). MicroRNA-23a in Human Cancer: Its Roles, Mechanisms and Therapeutic Relevance. Cancers.

[43] Dombret H., Itzykson R. (2017). How and when to Decide between Epigenetic Therapy and Chemotherapy in Patients with AML. Hematol. Am. Soc. Hematol. Educ. Program..

[44] Pleyer L., Dohner H., Dombret H., Seymour J.F., Schuh A.C., Beach C.L., Swern A.S., Burgstaller S., Stauder R., Girschikofsky M. (2017). Azacitidine for Front-Line Therapy of Patients with AML: Reproducible Efficacy Established by Direct Comparison of International Phase 3 Trial Data with Registry Data from the Austrian Azacitidine Registry of the AGMT Study Group. Int. J. Mol. Sci..

[45] Pleyer L., Burgstaller S., Stauder R., Girschikofsky M., Sill H., Schlick K., Thaler J., Halter B., Machherndl-Spandl S., Zebisch A. (2016). Azacitidine Front-Line in 339 Patients with Myelodysplastic Syndromes and Acute Myeloid Leukaemia: Comparison of French-American-British and World Health Organization Classifications. J. Hematol. Oncol..

[46] Gordon J.E., Wong J.J., Rasko J.E. (2013). MicroRNAs in Myeloid Malignancies. Br. J. Haematol..

[47] Austin C.A., Lee K.C., Swan R.L., Khazeem M.M., Manville C.M., Cridland P., Treumann A., Porter A., Morris N.J., Cowell I.G. (2018). TOP2B: The First Thirty Years. Int. J. Mol. Sci..

[48] Delgado J.L., Hsieh C.M., Chan N.L., Hiasa H. (2018). Topoisomerases as Anticancer Targets. Biochem. J..

[49] Pourquier P., Takebayashi Y., Urasaki Y., Gioffre C., Kohlhagen G., Pommier Y. (2000). Induction of Topoisomerase I Cleavage Complexes by 1-Beta -D-Arabinofuranosylcytosine (Ara-C) in Vitro and in Ara-C-Treated Cells. Proc. Natl. Acad. Sci. USA.

[50] Song J.H., Kweon S.H., Kim H.J., Lee T.H., Min W.S., Kim H.J., Kim Y.K., Hwang S.Y., Kim T.S. (2012). High TOP2B/TOP2A Expression Ratio at Diagnosis Correlates with Favourable Outcome for Standard Chemotherapy in Acute Myeloid Leukaemia. Br. J. Cancer.

[51] Caraffini V., Geiger O., Rosenberger A., Hatzl S., Perfler B., Berg J.L., Lim C., Strobl H., Kashofer K., Schauer S. (2020). Loss of RAF Kinase Inhibitor Protein is Involved in Myelomonocytic Differentiation and Aggravates RAS-Driven Myeloid Leukemogenesis. Haematologica.

[52] Prochazka K.T., Pregartner G., Rucker F.G., Heitzer E., Pabst G., Wolfler A., Zebisch A., Berghold A., Dohner K., Sill H. (2019). Clinical Implications of Subclonal TP53 Mutations in Acute Myeloid Leukemia. Haematologica.

[53] Caraffini V., Perfler B., Berg J.L., Uhl B., Schauer S., Kashofer K., Ghaffari-Tabrizi-Wizsy N., Strobl H., Wolfler A., Hoefler G. (2018). Loss of RKIP is a Frequent Event in Myeloid Sarcoma and Promotes Leukemic Tissue Infiltration. Blood.

[54] Lal R., Lind K., Heitzer E., Ulz P., Aubell K., Kashofer K., Middeke J.M., Thiede C., Schulz E., Rosenberger A. (2017). Somatic TP53 Mutations Characterize Preleukemic Stem Cells in Acute Myeloid Leukemia. Blood.

[55] Zebisch A., Lal R., Muller M., Lind K., Kashofer K., Girschikofsky M., Fuchs D., Wolfler A., Geigl J.B., Sill H. (2016). Acute Myeloid Leukemia with TP53 Germ Line Mutations. Blood.

[56] Zebisch A., Cerroni L., Beham-Schmid C., Sill H. (2003). Therapy-Related Leukemia Cutis: Case Study of an Aggressive Disorder. Ann. Hematol..

[57] Arber D.A., Orazi A., Hasserjian R., Thiele J., Borowitz M.J., Le Beau M.M., Bloomfield C.D., Cazzola M., Vardiman J.W. (2016). The 2016 Revision to the World Health Organization Classification of Myeloid Neoplasms and Acute Leukemia. Blood.

[58] Milewska M., Romano D., Herrero A., Guerriero M.L., Birtwistle M., Quehenberger F., Hatzl S., Kholodenko B.N., Segatto O., Kolch W. (2015). Mitogen-Inducible Gene-6 Mediates Feedback Inhibition from Mutated BRAF Towards the Epidermal Growth Factor Receptor and Thereby Limits Malignant Transformation. PLoS ONE.

[59] Zebisch A., Wolfler A., Fried I., Wolf O., Lind K., Bodner C., Haller M., Drasche A., Pirkebner D., Matallanas D. (2012). Frequent Loss of RAF Kinase Inhibitor Protein Expression in Acute Myeloid Leukemia. Leukemia.

[60] Auner H.W., Zebisch A., Schimek M.G., Bodner C., Hiden K., Linkesch W., Haas O.A., Beham-Schmid C., Sill H. (2004). High Expression of the Sister-Chromatid Separation Regulator and Proto-Oncogene hSecurin Occurs in a Subset of Myeloid Leukaemias but is Not Implicated in the Pathogenesis of Aneuploidy. Leukemia.

[61] Zebisch A., Haller M., Hiden K., Goebel T., Hoefler G., Troppmair J., Sill H. (2009). Loss of RAF Kinase Inhibitor Protein is a Somatic Event in the Pathogenesis of Therapy-Related Acute Myeloid Leukemias with C-RAF Germline Mutations. Leukemia.

[62] Zebisch A., Staber P.B., Delavar A., Bodner C., Hiden K., Fischereder K., Janakiraman M., Linkesch W., Auner H.W., Emberger W. (2006). Two Transforming C-RAF Germ-Line Mutations Identified in Patients with Therapy-Related Acute Myeloid Leukemia. Cancer Res..

[63] Gao J., Aksoy B.A., Dogrusoz U., Dresdner G., Gross B., Sumer S.O., Sun Y., Jacobsen A., Sinha R., Larsson E. (2013). Integrative Analysis of Complex Cancer Genomics and Clinical Profiles using the cBioPortal. Sci. Signal..

[64] Cerami E., Gao J., Dogrusoz U., Gross B.E., Sumer S.O., Aksoy B.A., Jacobsen A., Byrne C.J., Heuer M.L., Larsson E. (2012). The cBio Cancer Genomics Portal: An Open Platform for Exploring Multidimensional Cancer Genomics Data. Cancer Discov..

[65] Ruopp M.D., Perkins N.J., Whitcomb B.W., Schisterman E.F. (2008). Youden Index and Optimal Cut-Point Estimated from Observations Affected by a Lower Limit of Detection. Biom. J..

