# Supplementary material for: Increased Expression of Micro-RNA-23a Mediates Chemoresistance to Cytarabine in Acute Myeloid Leukemia
Source: Cancers (Basel). 2020 Feb 20;12(2):496. doi: 10.3390/cancers12020496 (PMC7072365; doi:10.3390/cancers12020496)
Supplement: Supplementary file 1 [file cancers-12-00496-s001.pdf]

cancers

Article

Increased Expression of Micro–RNA–23a Mediates Chemoresistance to Cytarabine in Acute Myeloid Leukemia

Increased Expression of Micro-RNA-23a Mediates Chemoresistance to Cytarabine in Acute Myeloid Leukemia

Stefan Hatzl [1], Bianca Perfler [1], Sonja Wurm [1], Barbara Uhl [1], Franz Quehenberger [2], Susanne Ebner [3], Jakob Troppmair [3], Andreas Reinisch [1], Albert Wölfler [1], Heinz Sill [1] and Armin Zebisch [1,4,*]

1 Division of Hematology, Medical University of Graz, Auenbruggerplatz 38, 8036 Graz, Austria; stefan.hatzl@medunigraz.at (S.H.); bianca.perfler@medunigraz.at (B.P.); wurm.sonja1@gmail.com (S.W.); barbara.uhl@medunigraz.at (B.U.); a.reinisch@medunigraz.at (A.R.);albert.woelfler@medunigraz.at (A.W.); heinz.sill@medunigraz.at (H.S.)

2 Institute for Medical Informatics, Statistics and Documentation, Medical University of Graz, 8036 Graz, Austria; franz.quehenberger@medunigraz.at

3 Daniel Swarovski Research Laboratory, Department of Visceral, Transplant and Thoracic Surgery, Medical University of Innsbruck, 6020 Innsbruck , Austria; susanne.ebner@i-med.ac.at (S.E.); jakob.troppmair@i-med.ac.at (J.T.)

4 Otto-Loewi-Research Center for Vascular Biology, Immunology and Inflammation, Division of Pharmacology, Medical University of Graz, Universitätsplatz 4, 8010 Graz, Austria

* Correspondence: armin.zebisch@medunigraz.at; Tel.: +43-316-385-80259; Fax: +43-316-385-14087

Received: 28 January 2020; Accepted: 18 February 2020; Published: date

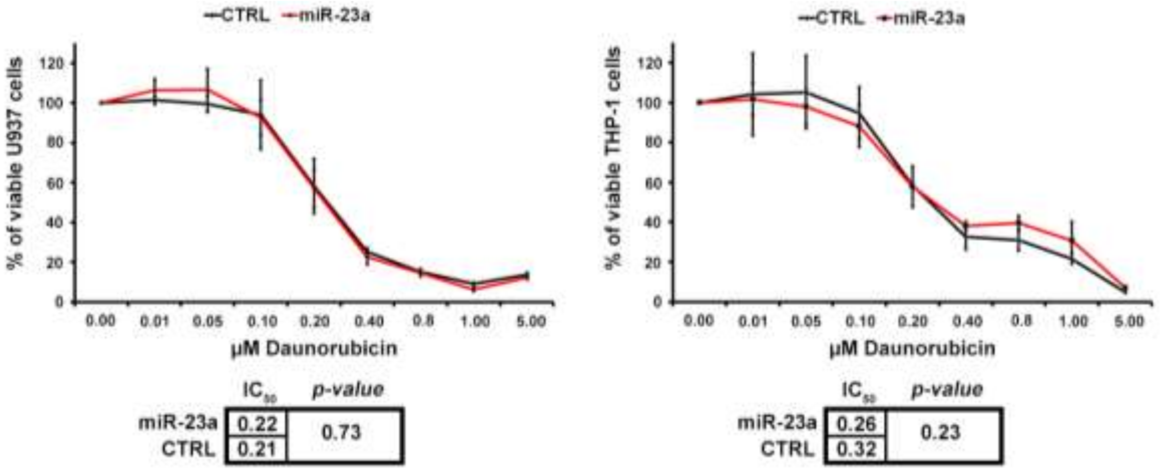

Figure S1. Sensitivity to daunorubicin after miR–23a modulation in AML cell lines. MTT cytotoxicity assays in U937 and THP–1 AML cells after incubation with daunorubicin. miR–23a denotes transduction with a miR–23a overexpression construct, CTRL denotes transduction with an empty control vector.

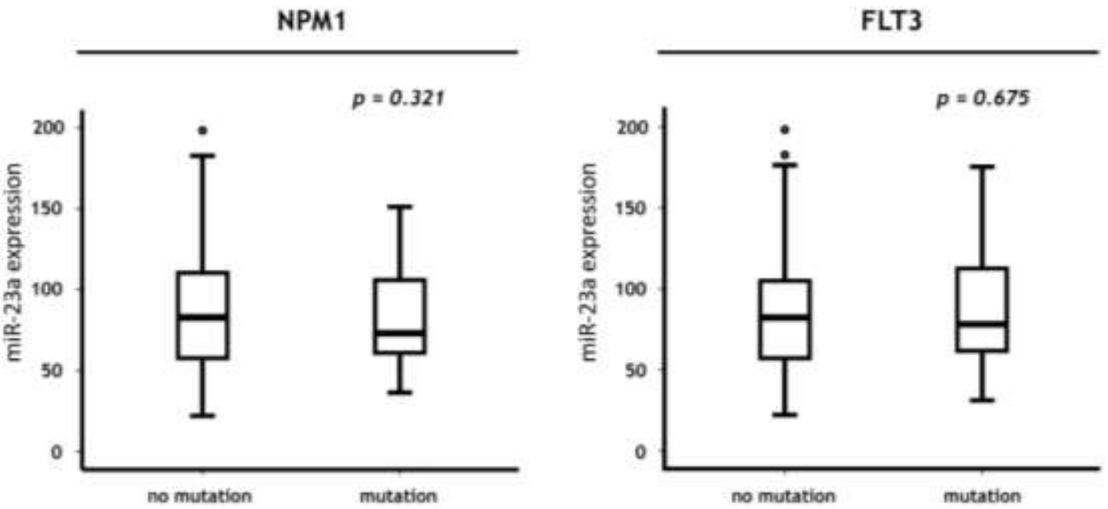

Figure S2. Expression of miR–23a does not correlate with *NPM1* and/or *FLT3* mutation status in AML. Box plots showing the correlation between miR–23a and the mutation status of *NPM1* and *FLT3*, respectively. miR–23a expression values are displayed as log–transformed miRNA–sequencing results. Data were downloaded from the TCGA AML cohort (n = 146) [1]. *NPM1* mutations were reported in 46/146 (32)% of all samples, *FLT3* in 41/146 (28%) of all cases.

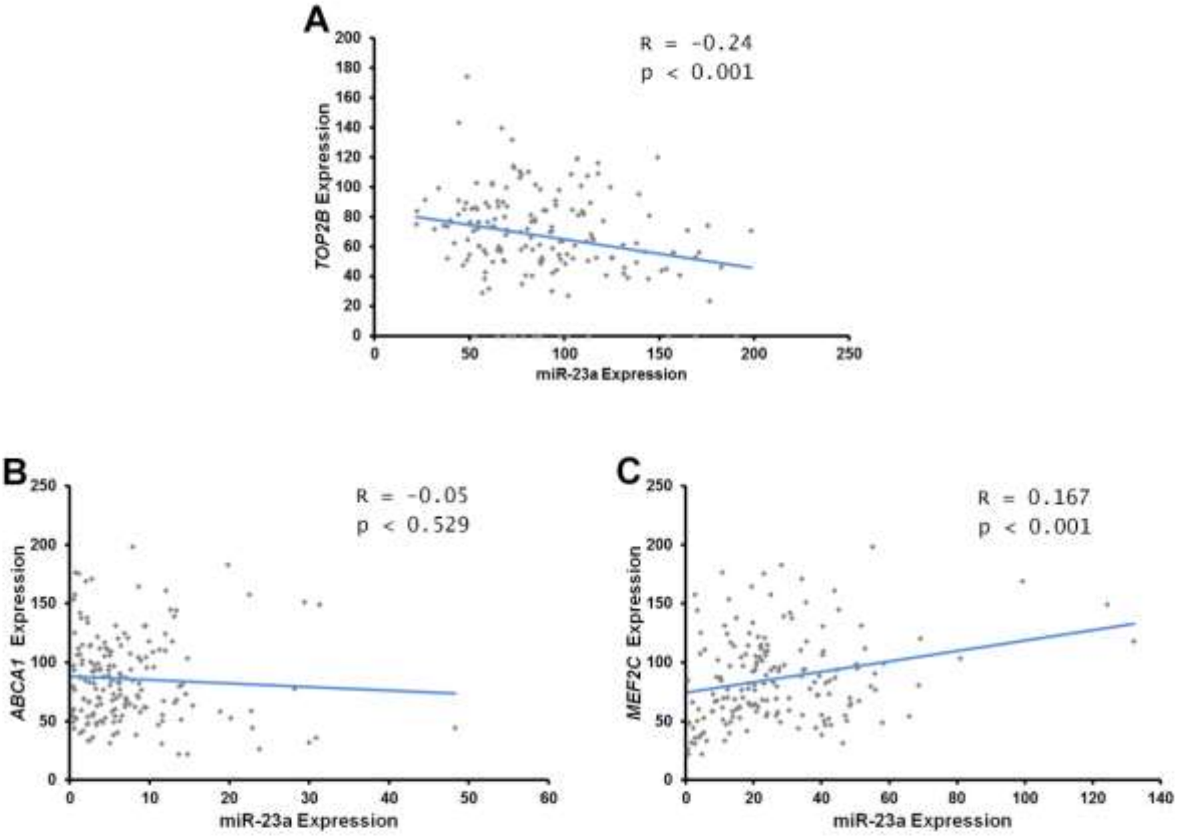

Figure S3. Increased expression of miR–23a correlates with decreased expression of TOP2B in AML. Scatter plots showing the correlation between miR–23a and *TOP2B* (A), *ABCA1* (B), as well as *MEF2C* mRNA (C). Expression values are displayed as RNA Sequencing V2 RSEM expression values for mRNAs (depicted at the y–axes), and as miRNA–sequencing expression values for miR–23a (depicted at the x–axes). Data were downloaded from the TCGA AML cohort (n = 173) [1].

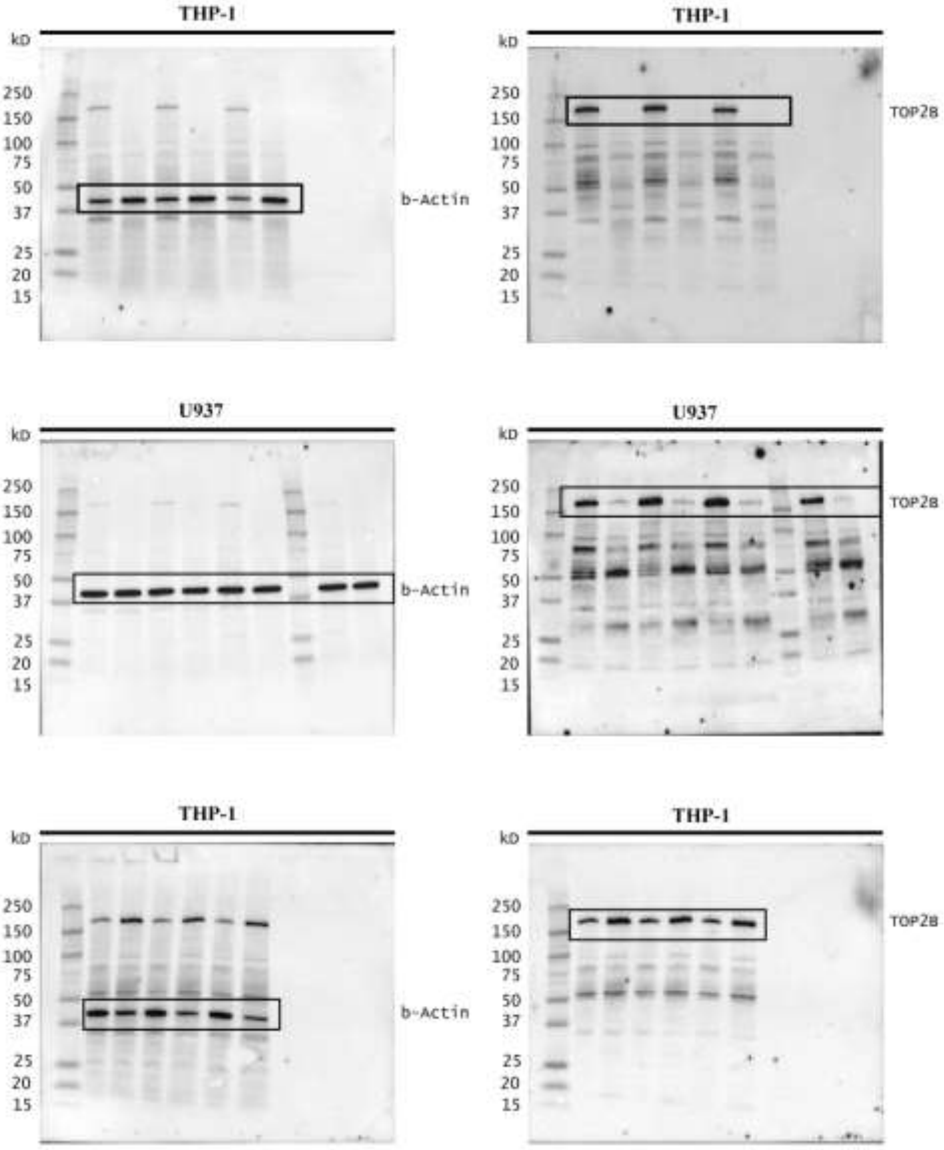

Figure S4. Uncropped Western Blots.

Table S1. Patient characteristics of the 24 paired AML specimens obtained at diagnosis and primary chemorefractory/relapsed disease. Pat., patient–number; WBC, white blood cells; f, female; m, male; G/L, giga per liter; LDH, lactate dehydrogenase; U/L, units per liter; ELN, European Leukemia Net risk stratification; FAB, French–American.British classification; HD, high–dose; AraC, cytarabine; HSCT, hematopoietic stem cell transplantation.

Pat.	Age	Gender	WBC (G/L)	Marrow Blasts (%)	LDH (U/L)	Karyotype	ELN	FAB	Therapy
AML 1	59	f	110	95	910	45,XX,t(2;3)(p23;q26),–7	adverse	M4	"7+3" + HD–AraC
AML 2	58	f	143	95	301	46,XX	favorable	M1	"7+3" + HD–AraC
AML 3	47	f	44.95	90	379	46,XX	favorable	M5	"7+3" + HD–AraC
AML 4	38	f	2.379	80	443	46,XX	intermediate	M2	"7+3" + HD–AraC
AML 5	38	f	186.12	95	2002	46,XX	intermediate	M4	"7+3" + HD–AraC+HSCT
AML 6	44	m	6.34	40	2101	46,XY	intermediate	M5	"7+3" + HD–AraC+HSCT
AML 7	21	m	0.78	50	5774	46,XY,t(6;11)(q27;q23)	intermediate	M5	"7+3" + HD–AraC+HSCT
AML 8	49	f	107	90	559	47,XX	intermediate	M0	"7+3"
AML 9	48	m	178	100	886	46,XY	intermediate	M2	"7+3" + HD–AraC
AML 10	54	m	49.24	80	1650	44~45,XY,–5,–7,–10,+2mar	adverse	M2	"7+3"
AML 11	54	m	24.78	70	313	46,XY	intermediate	M2	"7+3" + HD–AraC+HSCT
AML 12	49	m	2.01	80	203	46,XY	adverse	M1	"7+3"
AML 13	41	f	91	90	813	46,XX	favorable	M4	"7+3"
AML 14	65	f	40.73	90	393	46.XX	favorable	M4	"7+3" + HD–AraC
AML 15	68	m	30.85	95	786	45~47,XY,der(7)t(7;11)(p13;q13)del(7)(q31)	adverse	M0	"7+3" + HD–AraC
AML 16	58	f	60.53	95	622	49,XX,+6,+8,+22	adverse	M4	"7+3" + HD–AraC
AML 17	52	m	6,1	80	360	46.XY	intermediate	M4	"7+3" + HD–AraC+HSCT
AML 18	44	m	36.97	95	2041	46~48,XY,+8,ins(10;11)(p12;q23),+19	adverse	M0	"7+3" + HD–AraC
AML 19	55	f	234	95	883	46,XX	intermediate	M4	"7+3" + HD–AraC
AML 20	59	m	10.85	90	419	46~50,XY,+8, t(10;11)+13,+14,+19	adverse	M5	"7+3" + HD–AraC+HSCT
AML 21	46	f	64.87	95	498	46,XX	intermediate	M4	"7+3" + HD–AraC+HSCT
AML 22	66	f	3.85	90	288	46,XX	favorable	M1	"7+3" + HD–AraC
AML 23	67	m	12	85	213	46,XX, del(16)	favorable	M1	"7+3" + HD–AraC
AML 24	34	f	5.87	80	209	46,XX	intermediate	M2	"7+3" + HD–AraC+HSCT

Table S2. Patient characteristics of the 146 AML patients analyzed via the TCGA [1]. Patients were categorized according to their miR–23a expression as outlined in the main manuscript. Note that the exact time–point of allo–SCT was not available, therefore respective censoring in survival analyses was not possible. Details of risk stratification are outlined in the original TCGA publication [1]. WBC, white blood cells; BM, bone marrow; PB, peripheral blood; SCT, stem cell transplantation; CR, complete remission.

Variable	*n* (% miss.)	Overall (*n* = 146)	miR–23a high (*n* = 112)	miR–23a low (*n* = 34)	*p–value**
**Demographic variables**					
Age (years)	146 (0%)	51 (18–81)	51 (18–81)	51 (21–77)	0.989
Gender (female)	146 (0%)	67 (45%)	51 (45%)	16 (47%)	0.876
**Leukemia characteristics**					
WBC (per µL)	146 (0%)	37.8 (0.1–298.4)	47.2 (4.0–298.4)	4.8 (0.1–27.1)	**0.001**
BM–Blast count (%)	146 (0%)	72 (30–100)	72 (30–100)	72 (37–95)	0.972
PB–Blast count (%)	145 (0.6%)	42 (0–98)	43 (0–98)	40 (0–90)	0.427
**Risk stratification**					
Molecular Risk (points)	145 (0.6%)	2 (1–3)	2 (1–3)	2 (1–3)	0.402
Cytogenetic Risk (points)	144 (1.2%)	2 (1–3)	2 (1–3)	2 (1–3)	0.273
**Transplant characteristics**					
Allogenic SCT (%)	146 (0%)	70 (48%)	54 (48%)	16 (47%)	0.392
Allogenic SCT in 1st CR (%)	146 (0%)	47 (32%)	39 (35%)	8 (27%)	0.217

Table 3. TOP50 hits of the in–silico screening for potential miR–23a target genes by employing the miR–walk 2.0 algorithm ([2]; http://zmf.umm.uni–heidelberg.de/apps/zmf/mirwalk2/). The column "Prediction" demonstrates the results of the seven target prediction tools included in this algorithm (miRWalk; Microt4, http://diana.imis.athena–innovation.gr/DianaTools; miRanda, http://microrna.org/; miRMap, https://mirmap.ezlab.org/; miRNAMap, http://mirnamap.mbc.nctu.edu.tw/; RNAhybrid, https://bio.tools/rnahybrid; Targetscan, http://www.targetscan.org/vert_72/. 7/7 indicates that a specific gene has been identified as a potential miR–23a target in all seven target prediction machines. The column "PMID" presents the results of a PubMed–based literature search. The respective gene was entered along with "AML" or "Acute myeloid leukemia", and "therapeutic resistance" or "chemoresistance". Matches are displayed with the respective PubMed ID and highlighted in red, no match is indicated as x.

Gene	Prediction	PMID	Gene	Prediction	PMID	Gene	Prediction	PMID
CCDC6	7/7	x	*WNK3*	7/7	x	*DICER1*	7/7	x
TENM1	7/7	x	*PTCH1*	7/7	x	*FNTA*	7/7	x
METAP1	7/7	x	*VGLL2*	7/7	x	*SUCO*	7/7	x
CAPN6	7/7	x	*TERF2*	7/7	x	*LDHB*	7/7	x
TENM4	7/7	x	*DNAJC6*	7/7	x	*SLC39A10*	7/7	x
FOXA1	7/7	x	*SEC24A*	7/7	x	*NAP1L1*	7/7	x
MSL2	7/7	x	*ESYT1*	7/7	x	*KLHL15*	7/7	x
MRC1	7/7	x	*DLX1*	7/7	x	*RXRG*	7/7	x
RBM25	7/7	x	*BBS9*	7/7	x	*LIN9*	7/7	x
PPP1CB	7/7	x	*ITGB8*	7/7	x	*KDM6A*	7/7	x
RNF38	7/7	x	*MYNN*	7/7	x	*LRPPRC*	7/7	x
TADA2A	7/7	x	*MYH2*	7/7	x	*UHRF1BP1L*	7/7	x
CUL3	7/7	x	*VCPIP1*	7/7	x	*CACNB2*	7/7	x
SEC23A	7/7	x	*RAD23B*	7/7	x	*SMPX*	7/7	x
NEDD4L	7/7	x	*CNOT6L*	7/7	x	*GALNT1*	7/7	x
RUNX2	7/7	x	*KLF10*	7/7	x	*BRWD1*	7/7	x
FOXP1	7/7	x	*ABI1*	7/7	x	*MCM6*	7/7	x
HOXC11	7/7	x	*ZHX1*	7/7	X	*NLGN4X*	7/7	x
LGR4	7/7	x	*ABCA1*	7/7	26463638	*NTS*	7/7	x
MYH1	7/7	x	*MED13L*	7/7	x	*ZIC4*	7/7	x
RBM26	7/7	x	*EGR2*	7/7	x	*SLC20A1*	7/7	x
PPP6C	7/7	x	*HOOK2*	7/7	x	*SPOPL*	7/7	x
RDH10	7/7	x	*IPO5*	7/7	x	*VSNL1*	7/7	x
TCF20	7/7	x	*MYO5C*	7/7	x	*ZMYM2*	7/7	x
SLC4A4	7/7	x	*MYH4*	7/7	x	*GPR64*	7/7	x
CELF2	7/7	x	*NAA15*	7/7	x	*POGZ*	7/7	x
WDR7	7/7	x	*RPS6KA3*	7/7	x	*CALCR*	7/7	x
CLK3	7/7	x	*MAGI3*	7/7	x	*ZNF521*	7/7	x
ARFIP1	7/7	x	*TOP2B*	7/7	22627319	*GAP43*	7/7	x
HOXD10	7/7	x	*COL4A3BP*	7/7	x	*RC3H2*	7/7	x
AMBRA1	7/7	x	*ZBTB1*	7/7	x	*MEF2C*	7/7	29431698
MYH2	7/7	x	*ADH5*	7/7	x	*ARRDC3*	7/7	x
SPTBN1	7/7	x	*TTC7B*	7/7	x	*POU4F2*	7/7	x

Table S4. Gene signatures of miR–23a and *TOP2B* in comparison to the previously published LSC and OXPHOS signatures [3,4]. No overlaps exist between these signatures. miR–23a and *TOP2B* signatures have been established as described in the main manuscript.

miR–23a DOWN	miR–23a UP	*TOP2B* Interact	LSC	OXPHOS DOWN	OXPHOS UP
BBS9	*CD34*	*CBX8*	*NPAL2*	*C9orf153*	*ANGPT2*
C7ORF61	*COL4A3BP*	*CTCF*	*TRAF3IP2*	*CGRPR*	*ARHGEF12*
CHD6	*EGR2*	*DDX18*	*PPP1R10*	*CD200*	*BCL6*
DHRS13	*GMFG*	*DDX31*	*NF1*	*CMKLR1*	*BIRC3*
DNAJC6	*IL6R*	*HDGF*	*FLJ13197*	*CPA6*	*C3*
FAM102A	*KRT80*	*HMGA1*	*ABCG1*	*CYP51A1*	*CA1*
FGFR1	*LUZP1*	*LRIF1*	*CLN5*	*DCSTAMP*	*CCL23*
GPRIN2	*MEAK7*	*MORC2*	*LRRC8B*	*DHCR24*	*CDK15*
HSD11B1L	*MEF2C*	*NIPBL*	*FRMD4B*	*DPP10*	*CHRNA6*

KDF1	*PPP1CB*	*PDS5A*	*ZFP30*	*ERMN*	*CLC*
LDHB	*SEC23A*	*PDS5B*	*C17orf86*	*EYS*	*CSF1*
LIPT1	*SNX21*	*PHF2*	*C16orf5*	*FAM160A1*	*CSF2RB*
MAGI2	*SPOPL*	*RAD21*	*TGIF2*	*FAM47E*	*CXCL10*
METAP1	*UHRF1BP1L*	*RRP15*	*RABGAP1*	*FLJ45983*	*CXCL11*
MSL2	*VCPIP1*	*SDAD1*	*PPIG*	*GALNT12*	*DDX60L*
MYH1		*SMC1A*	*GPR56*	*GUCY1A3*	*EFHC2*
NAA16		*SRBD1*	*EIF2S3*	*IGJ*	*ENPP3*
NAP1L1		*STAG1*	*NAB1*	*IGLL3P*	*ENTPD1*
PEBP1		*STAG2*	*LRRC61*	*IL2RA*	*EPSTI1*
PHKG1		*TOP1*	*ATP1B1*	*IL3RA*	*EPX*
PI16		*TOP2A*	*ZNF500*	*INSIG1*	*FAM198B*
PLIN4		*YY1*	*CSDE1*	*KCNA7*	*FCGR1A*
PRRT1		*ZNF362*	*C2CD2*	*KCNK17*	*FCGR1B*
PTCH1		*ZNF451*	*PAQR6*	*KIAA0125*	*FNBP1L*
RBM25		*ZNF512*	*FAM119B*	*KRT5*	*GATM*
SERHL2			*ARPP–19*	*LEPREL1*	*GMPR*
TENM4			*SETDB1*	*MAP1A*	*GPR85*
TOP2B			*ZBTB39*	*MIR126*	*HBB*
UBASH3A			*RBPMS*	*MMP2*	*HBD*
VHLL			*SLC9A7*	*MYO5C*	*HDC*
ZMYM2			*ARL3*	*MZB1*	*HERC5*
ZNF831			*ZNF304*	*PLA2G10*	*HGF*
			LOC552889	*PRDM7*	*HNRPLL*
			VGLL4	*PTPN20A*	*HPGDS*
			UBR5	*RAB20*	*IFI44L*
			PTCD2	*S100A3*	*IL1RL1*
			CRKRS	*SCARNA9*	*IL6ST*
			IQGAP2	*SLA2*	*ITGB3*
			PLCH1	*SLC6A5*	*JMY*
			ARFGEF1	*SNORA65*	*KEL*
			MAP3K7	*SNORD116–26*	*LTBP1*
			PNPLA4	*SNORD60*	*MED12L*
				SPNS3	*MIR222*
				STARD4	*MNDA*
				STARD6	*NPL*
				STAT4	*P2RY13*
				TTC39A	*PARM1*
				XKR3	*PPBP*
				ZMAT4	*PRG2*
				ZNF283	*RHAG*
				ZNF737	*SERPINB10*
					SERPINB2
					SESN3
					SLC10A5
					SLC1A3
					SLC22A15
					SLC27A6
					SLCO4C1
					SLFN5
					SNORA36A
					ST8SIA6
					TC2N
					TLE1
					TLR4
					TNFSF10
					TNIK
					VNN2
					ZFP36L1

Table S5. Univariate Cox regression analysis for EFS and OS for the miR–23a and *TOP2B* gene signatures. The influence on prognosis was calculated as previously described [3,4]. In more detail, z–scores were downloaded from the TCGA [1] for all genes of each signature. Then, the sum of all z–scores was calculated for each patient and subsequently used as value for calculation of prognosis. Note that even the statistical significance of miR–23a DOWN could not be validated in a multivariate cox proportional hazards model after adjusting for at least one of the univariable established predictors of survival (WBC, age, cytogenetic risk and molecular risk). EFS, event–free survival; OS, overall survival; CI, confidence interval; WBC, white blood cell count.

Parameter	Variable	Hazard ratio	95% CI	*p*–value
	miR–23a DOWN	1.21	1.005–1.410	*0.003*
OS	miR–23a UP	1.00	0.99–1.010	*0.097*
	TOP2B Interact	0.98	0.910–1.021	*0.420*
	miR–23a DOWN	1.15	1.051–1.243	*0.005*
EFS	miR–23a UP	1.02	0.985–1.045	*0.500*
	TOP2B Interact	0.92	0.850–1.021	*0.569*

Table 6. Primer sequences and/or ordering information used for qPCR analyses.

Gene	forward	reverse
TOP2B	AGCCATTGACGCAGTTCATGT	CCTGGCACAAAGGTAACCTCC
B2M	CGCTCCGAGATGCATGTG	TTGGCTGGCAGTCCTTTAGG
GUSB	CCTGAAGGTGGCTGTGAAGATG	GCTCCCAGAAGGTTGACGATG
SNORD44	Qiagen, Cat# MS00007518	
RNU6	Qiagen, Cat# MS00003740	
miR–23a	Qiagen, Cat# MS00031633	

Reference

1. Cancer Genome Atlas Research Network. Genomic and Epigenomic Landscapes of Adult De Novo Acute Myeloid Leukemia. *N. Engl. J. Med.* **2013**, *368*, 2059–2074, doi:10.1056/NEJMoa1301689.
2. 2 Dweep H and Gretz N. miRWalk2.0: A comprehensive atlas of microRNA–target interactions. *Nat Methods* **2015**, *12*, 697.
3. Eppert. K.; Takenaka, K.; Lechman, E.R.; Waldron, L.; Nilsson, B.; van Galen, P.; Metzeler, K.H.; Poeppl, A.; Ling, V.; Beyene, J.; et al. Stem cell gene expression programs influence clinical outcome in human leukemia. *Nat. Med.* **2011**, *17*, 1086
4. Farge, T.; Saland, E.; de Toni, F.; Aroua, N.; Hosseini, M.; Perry, R.; Bosc, C.; Sugita, M.; Stuani, L.; Fraisse. M.; et al. Chemotherapy–resistant human acute myeloid leukemia cells are not enriched for leukemic stem cells but require oxidative metabolism. *Cancer Discov.* **2017**, *7*, 716.
